# Supplementary material for: Comparative genomics provides new insights into the diversity, physiology, and sexuality of the only industrially exploited tremellomycete: Phaffia rhodozyma
Source: BMC Genomics. 2016 Nov 9;17:901. doi: 10.1186/s12864-016-3244-7 (PMC5103461; doi:10.1186/s12864-016-3244-7)
Supplement: Additional file 6: — List of orphan genes with links to PFAM (related to Additional file 1: Table S1). (ZIP 1428 kb) [file 12864_2016_3244_MOESM6_ESM.zip › BLAST_HTML_FTR/G01021_P.html]

BLAST Search Results


```
BLASTP 2.2.27+


Reference:
Stephen F. Altschul, Thomas L. Madden, Alejandro A. Schäffer,
Jinghui Zhang, Zheng Zhang, Webb Miller, and David J. Lipman (1997),
"Gapped BLAST and PSI-BLAST: a new generation of protein database
search programs", Nucleic Acids Res. 25:3389-3402.


Reference for
composition-based statistics:
Alejandro A. Schäffer, L. Aravind, Thomas L. Madden, Sergei
Shavirin, John L. Spouge, Yuri I. Wolf, Eugene V. Koonin, and
Stephen F. Altschul (2001), "Improving the accuracy of PSI-BLAST
protein database searches with composition-based statistics and
other refinements", Nucleic Acids Res. 29:2994-3005.


Database: nr
           71,551,133 sequences; 26,053,659,533 total letters


Query= G01021_P

Length=340
                                                                      Score     E
Sequences producing significant alignments:                          (Bits)  Value

emb|CDZ97198.1|  hypothetical protein [Xanthophyllomyces dendrorh...   686    0.0  
dbj|BAR48642.1|  F5/8 type C domain protein [Tannerella forsythia...  43.1    0.36 
dbj|BAR51276.1|  F5/8 type C domain protein [Tannerella forsythia...  43.1    0.36 


 >emb|CDZ97198.1| hypothetical protein [Xanthophyllomyces dendrorhous]
Length=339

 Score =  686 bits (1769),  Expect = 0.0, Method: Compositional matrix adjust.
 Identities = 339/339 (100%), Positives = 339/339 (100%), Gaps = 0/339 (0%)

Query  1    MSNAAPHSHLEDHPSTIKRRVDFGARPRKLLTKRSFKTSISDNNENESLSSSSGGAVWAK  60
            MSNAAPHSHLEDHPSTIKRRVDFGARPRKLLTKRSFKTSISDNNENESLSSSSGGAVWAK
Sbjct  1    MSNAAPHSHLEDHPSTIKRRVDFGARPRKLLTKRSFKTSISDNNENESLSSSSGGAVWAK  60

Query  61   GRKCMKRSEGSTANRICKSLQTHSSSPPLSTANNNLSRRSEHYDFQLNHDSDHDFDGCSS  120
            GRKCMKRSEGSTANRICKSLQTHSSSPPLSTANNNLSRRSEHYDFQLNHDSDHDFDGCSS
Sbjct  61   GRKCMKRSEGSTANRICKSLQTHSSSPPLSTANNNLSRRSEHYDFQLNHDSDHDFDGCSS  120

Query  121  SSTKGHPIRPNFFLHRSSSTPLLSLSTASLQTLNERVATQETMFLNSFLTLQPMSLERSN  180
            SSTKGHPIRPNFFLHRSSSTPLLSLSTASLQTLNERVATQETMFLNSFLTLQPMSLERSN
Sbjct  121  SSTKGHPIRPNFFLHRSSSTPLLSLSTASLQTLNERVATQETMFLNSFLTLQPMSLERSN  180

Query  181  KPTLFPSDRSPRVEKDTVPTAPGTANSTASFFSTSTESSTVLTPSDYLFSFQQTTNLSGA  240
            KPTLFPSDRSPRVEKDTVPTAPGTANSTASFFSTSTESSTVLTPSDYLFSFQQTTNLSGA
Sbjct  181  KPTLFPSDRSPRVEKDTVPTAPGTANSTASFFSTSTESSTVLTPSDYLFSFQQTTNLSGA  240

Query  241  CSDIDKSDVMDLDLDLPNLSVSSSSTHSSSWAELEGGTEPTEPDEEDEDDLIVLDESFGL  300
            CSDIDKSDVMDLDLDLPNLSVSSSSTHSSSWAELEGGTEPTEPDEEDEDDLIVLDESFGL
Sbjct  241  CSDIDKSDVMDLDLDLPNLSVSSSSTHSSSWAELEGGTEPTEPDEEDEDDLIVLDESFGL  300

Query  301  EGCFGKVELMDELSMMMKRAHLPKPSFSDEDLDPGELFD  339
            EGCFGKVELMDELSMMMKRAHLPKPSFSDEDLDPGELFD
Sbjct  301  EGCFGKVELMDELSMMMKRAHLPKPSFSDEDLDPGELFD  339


>dbj|BAR48642.1| F5/8 type C domain protein [Tannerella forsythia 3313]
Length=684

 Score = 43.1 bits (100),  Expect = 0.36, Method: Compositional matrix adjust.
 Identities = 43/142 (30%), Positives = 64/142 (45%), Gaps = 4/142 (3%)

Query  164  FLNSFLTLQPMSLERSNKPTLFPSDRSPRVEKDTVPTAPGTANSTASFFSTSTESSTVLT  223
            FL++   L   ++E    P L    R  R EKD V   PG  NS   + + +++ S   T
Sbjct  466  FLDARGPLCINNVEAFLAPALMVEPRIVRNEKDEVKILPGDKNSVVYYTTDNSQPSLEST  525

Query  224  PSDYLFSFQQTTNLSGACSD--IDK-SDVMDLDLDLPNLSVSSSSTHSSSWAELEGGTEP  280
              D+ FSF Q   +  AC D   DK S V   +LD+P  S   +    ++  + +G +  
Sbjct  526  RYDHPFSFAQKGRIKAACYDATFDKWSPVTTAELDIPASSYEMADEKMTALFDGDGNSLY  585

Query  281  TEPDEEDEDDLIVLDESFGLEG  302
            T P  E+    IVL E   + G
Sbjct  586  TLPKGEN-SLTIVLKEKATIAG  606


>dbj|BAR51276.1| F5/8 type C domain protein [Tannerella forsythia KS16]
Length=684

 Score = 43.1 bits (100),  Expect = 0.36, Method: Compositional matrix adjust.
 Identities = 43/142 (30%), Positives = 64/142 (45%), Gaps = 4/142 (3%)

Query  164  FLNSFLTLQPMSLERSNKPTLFPSDRSPRVEKDTVPTAPGTANSTASFFSTSTESSTVLT  223
            FL++   L   ++E    P L    R  R EKD V   PG  NS   + + +++ S   T
Sbjct  466  FLDARGPLCINNVEAFLAPALMVEPRIVRNEKDEVKILPGDKNSVVYYTTDNSQPSLEST  525

Query  224  PSDYLFSFQQTTNLSGACSD--IDK-SDVMDLDLDLPNLSVSSSSTHSSSWAELEGGTEP  280
              D+ FSF Q   +  AC D   DK S V   +LD+P  S   +    ++  + +G +  
Sbjct  526  RYDHPFSFAQKGRIKAACYDATFDKWSPVTTAELDIPASSYEMADEKMTALFDGDGNSLY  585

Query  281  TEPDEEDEDDLIVLDESFGLEG  302
            T P  E+    IVL E   + G
Sbjct  586  TLPKGEN-SLTIVLKEKATIAG  606


Lambda      K        H        a         alpha
   0.312    0.126    0.361    0.792     4.96 

Gapped
Lambda      K        H        a         alpha    sigma
   0.267   0.0410    0.140     1.90     42.6     43.6 

Effective search space used: 2882143867050


  Database: nr
    Posted date:  Sep 23, 2015 12:05 AM
  Number of letters in database: 26,053,659,533
  Number of sequences in database:  71,551,133


Matrix: BLOSUM62
Gap Penalties: Existence: 11, Extension: 1
Neighboring words threshold: 11
Window for multiple hits: 40
```
